# How Can Poetry Support the Understanding of Psychotic Experiences? – A Conceptual Review

**Published:** 2020

**Authors:** Mark Pearson, Stefan Rennick-Egglestone, Gary Winship

**Affiliations:** 1 School of Health Sciences, University of Nottingham, Nottingham, United Kingdom; 2 School of Social Sciences, University of Nottingham, Nottingham, United Kingdom

**Keywords:** Medicine, Mental Health, Humanities, Qualitative Research, Medication Adherence, Psychosis

## Abstract

**Objective:**

The therapeutic application of poetry for those who have experienced psychosis remains under researched and potentially undervalued. This paper presents a conceptual review exploring the relationship between poetry and psychosis, based on a synthesis of existing literature.

**Research Design and Methods:**

The review identified papers from a range of sources and disciplines. Initial searches were undertaken using databases CINAHL, PsycINFO and ASSIA; this search was then followed up with a library search for key texts and a further search for associated grey literature involving exploring blogs and conference presentations. The data was then synthesized based on methods from both narrative review and thematic analysis to generate a conceptual framework.

**Results:**

The results reveal a conceptual framework comprised of three domains: i) psychotic language as meaningful poetics, ii) poetry as an expression of psychosis and iii) poetic exchange as therapeutic practice. The conceptual framework proposes that not only can psychosis be understood as meaningful poetics, but also that poetry may offer meaningful linguistic opportunities to aid the expression and narration of self and experiences. The potential for extending our understanding of the poetry in this way is analogous to forms of talking therapy, and this may be a base for extending understanding and communicative practice for a range of mental health professions.

**Conclusion:**

The conceptual framework suggests a novel understanding of psychosis in relation to poetry, moving away from traditional biomedical paradigms and placing importance upon individual narratives.

## Introduction

The concept of poetry as a therapeutic tool, originates from a convergence of several theoretical, practical and philosophical fields.^1^ However, one of the most fundamental aspects of poetry is its ability to enable people to connect with themselves.^2^ Poetry and poetic writing resonates with the way in which identity is shaped through narratives.^3^ This process has the potential for special interest in relation to the experience of psychosis, often characterized as a disturbed perception of one’s self within a personal, environmental and social context.^4, 5^ Whilst the association between psychosis and creative individuals, especially poets, was established in antiquity^6,7^ only limited research has been undertaken in this area.^8, 9^ Therefore, the role of poetry as a therapeutic tool for those experiencing psychosis remains under researched.

The suggestion that creative practices might hold merit is one that has interested those working in mental health for decades. The psychiatrist RD Laing spent much of his career trying to understand psychosis, finding academic writing to be inadequate and thus experimenting with forms of writing poetry from which to build theory.^10^ However, the use of poetry remains a significant divergence from the dominant biomedical treatments which rely heavily on pharmacological interventions. ^11^ Whilst there is evidence to suggest the efficacy of pharmacological treatments in reducing psychotic symptomology^12^, such interventions do not necessarily support the individual to understand or make meaning from their experiences.^13^ This process of meaning making is essential in achieving a stable sense of one’s identity, and is central to the notion of recovery within mental health, which refers to the process of achieving a meaningful and resilient life regardless of what might be considered symptoms or disabilities.^14^

## Research Design and Methods

It is the aim of conceptual reviews to explore variations in the understanding of phenomena, presenting concepts not as individual abstract entities, but as situated within a network of meanings.^15^ Therefore, this conceptual review did not seek to review all papers, but rather, due to the complexity of the topic, to explore the relationship between poetry and psychosis by searching and synthesizing a range of evidence from a variety of disciplines, ensuring safeguards were established to help prevent bias.^16^

The initial stage of the present review involved searching for literature using established databases. The researchers selected the databases CINAHL, PsycINFO and ASSIA in order to ensure a range of literature from health sciences, medicine, psychology and social sciences. One additional search was also undertaken within the journal of *Poetry Therapy* due to the specific focus of the journal and its significant relevance to the focus of the review. Identical search terms were used in all searches undertaken across the databases, utilizing the key words ‘psychosis OR psychotic disorder OR schizophrenia AND poetry OR poetry therapy OR poet*’. Once duplicates had been removed, the results were then initially filtered by excluding results that were not available in full text, in English or were not peer reviewed. Following this initial filtering of results, the abstracts of the remaining papers were studied and further exclusions were made based on relevance to the focus of the review. For example, some papers were excluded due to not relating to psychosis but rather focusing on other specialties such as learning disabilities or neurodiversity or nurse education.

The terms ‘psychosis’ and ‘psychotic disorder’ were chosen as these represent the transdiagnostic terms for a much larger group sub diagnoses.^17^ The transdiagnostic terms consider mental illness and diagnoses as existing on a spectrum thus encompassing the idiosyncratic symptoms, which might be experienced by individuals. Furthermore, the term psychosis is observed within contemporary diagnostic frameworks to reflect a range of mental health problems.^18^ The inclusion of schizophrenia followed the observation in that the majority of research in this field has focused on the diagnosis of schizophrenia, as opposed to other psychotic disorders.^17^ The terms poetry, poetry therapy and poet* were used to focus the search within the interests of this literature review.

Following the initial database search, a library search was undertaken, using an online search engine to identify texts associated with the emerging key theoretical concepts related to the topic. This included psychosis, semiotics, health humanities, creative practice, art therapy and psychotherapy. This data was then augmented by a further search for grey literature, initially involving searching the archives of keynote presentations delivered at the national association of poetry therapy annual conference. In order to enhance the breadth of the review, an online search was also conducted using internet search engine ‘google’ to find blogs and online forums. As search engines do not have equivalent searching intricacies to databases, it was not possible to repeat the database search with identical key words and inclusion/exclusion criteria. Therefore, a search for psychosis AND poetry was undertaken. The results from this search highlighted a series of blogs associated with both poetry and mental health. The content was primarily people sharing their poems accompanied by stories and background to the poems. As the work recognizes the individual experience of psychosis and poetry, it was relevant and important to include data available through blogs in order to gain some insight into the lived experience of psychosis in relation to poetry. Moreover, the knowledge gained from these blogs and forums is a distinctly different and as such can provide an insight online communities, adding new voices to academic discourse.^19^

The data collected through the review was subject to an inductive analytic process influenced by a narrative review approach^20^ and a thematic analysis.^21^ In accordance with the narrative review guidance, attention was given to not over combining concepts due to the risk of losing discrete concepts. Rather, the process focused on identifying the themes within the literature and exploring the relationships between these concepts. As with narrative reviews, the purpose of the results was to synthesize the evidence found within the review and present this in a way that represented the broad perspectives on the topic. Therefore, the initial synthesis was undertaken by MP, who identified the common theme and then synthesized into domains, which represent the elements of the conceptual framework. Once the initial domains had been developed the domains and the elements contained within each domain were discussed with two further analysts GW and SRE due to their expertise in health humanities, creative practice and narrative approaches. Following these discussions, the domains were then developed further through a process of discussion and deliberation regarding further sources of data which could be accessed to support the development of the framework. Furthermore, these discussions helped to refine the framework, defining the individual elements of the structure and identifying the distinct elements of each domain.

## Results

The following domains represent the conceptual framework generated following the synthesis of data gathered in the literature review. The framework consists of 3 domains: psychotic language as meaningful language, narratives, psychosis and Poetry, Psychotherapeutic practice and poetry.

The first of these components relates to the way in which meaning making can be undertaken and achieved when working with individuals who have experienced psychosis. The second component focused on the linguistic theory underpinning the way in which poetry might present new linguistic and opportunities to aid the expression and narration of self and experiences. Finally, the third component focuses on the way in which poetry is or could be used within psychotherapeutic clinical practice.

### Psychotic language as meaningful poetics

Psychotic speech has historically been viewed as unstable and representative of the chaotic thought disorder experienced by an individual experiencing psychosis.^23^ However, such as reductive hypothesis fails to consider that whilst people who have experienced psychosis often communicate in idiosyncratic linguistic terms, these terms which are rich in emotive content.^24^ The significance of acknowledging psychotic speech as an expression of meaningful communication is further emphasized when psychotic phenomena are observed not as a biomedical pathology, but as an expression of distress.^25–28^ An expression which is often communicated in response to profound trauma.^29–31^

Whilst previous research has attempted to explore psychotic speech, this is often with a focus on observing underlying pathologies.^32^ Much of the recent investigations in this area have focused on the potential of language features to align with specific diagnostic criteria.^33^ Whilst providing some interesting insight into lexical markers and potentially contributing some diagnostic orientation,^32^ this field of research offers little in the pursuit of understanding the psychotic experience or supporting those who have experienced psychosis to make meaning from their experiences.

Whereas, when the linguistic system is approached as the primary system through which individuals make sense and meaning of their experiences,^34^ those experiencing psychotic symptoms can be viewed, not as passive victims afflicted by a disorder, but rather as individuals who are engaged in an ongoing meaning making process.^26^ Unfortunately, a considerable barrier preventing individuals from completing this process is the manner in which psychotic speech is likely to be dismissed as incoherent within contemporary mental health clinical practice.^35^

Notions of coherence, referring to the quality of being logical, rational and reasonable are often qualities that are required within narratives whether those be personal or otherwise. Mandler^36^ suggests that the majority of stories within society, follow a similar plot line, in which one event is triggered by the next however, a narrative which seems unimaginable is likely to be experienced as incoherent and one which lacks the structural composition.^37^ It could be argued that any narrative which tries truly to reflect the complexity of life will be incoherent, as life itself is often incoherent.^38^ However, traditionally within psychiatric discourse chaotic narratives are viewed as an indicator of illness and disorder within the mind^39^ or a lack of education.^40^

McAdams^37^ asserts that the problem of coherence is ultimately perpetuated by the concern that one is not being understood. If the narrative that is being conveyed is not being understood, then the narrator is likely to recognize a futility in continuing the storytelling process, instead withdrawing from conversation.^41^ Equally, the sense of futility may also pervade the mind of the interlocutor within the conversation. The interlocutor often fails to understand what is being conveyed whilst simultaneously being highly cognisant of the emotive haemorrhaging experienced by the narrator.^42^ The result is an interlocutor oscillating between a position of confusion and care giving, with the likely result of both positions becoming ineffective due to the disorganized response of the interlocutor.

The lack of a shared philosophical vocabulary is a significant factor in the labelling of a narrative as incoherent. The notion of coherence requires accepted linguistic parameters to designate what is considered rational in relation to an accepted positivist perspective.^43^ However, such a literal approach to language fails to engage with abstract concepts, and narrating abstract concepts is often at the heart of understanding and narrating psychotic experiences. Therefore, there is the potential for expression to become restricted as individuals find themselves unable to express their experiences within socially accepted parameters.^43^

Interestingly, several of the characteristics within what might be considered incoherent speech, such as loosening of association, are observed within poetry. Furthermore, when observed within poetry, these features are not only viewed as coherent but are imbued with significant artistic and personal meaning.^24^ Therefore, it may be that poetry offers a medium through which psychosis and psychotic narratives can be understood. To accept and engage with the meaning expressed though poetry is not an exercise in striving for coherence, but rather represents an opportunity to engage with the incoherent and embrace the psychotic communication as another voice within the dialogue.^44^

Unfortunately, such an embrace of the incoherent is somewhat counter intuitive within dominant contemporary psychiatric paradigms. To work with incoherence is to work with uncertainty, and uncertainty is historically a topic which mental healthcare has struggled to reconcile, especially in the face of advances in other medical fields in which increasingly precise diagnoses can be generated.^45^ This ‘tyranny of certainty’ leads to the use of words becoming narrow and restrictive.^46^ However, Strauss^47^ described uncertainty theory as a way of being in which individual’s acknowledge their own lack of certainty in a given situation, emphasizing that they may be unsure where to look for answers to a particularly question, and, in turn, free themselves to question and explore. It is the acceptance of not knowing which enables meaningful broad inquisition, not restricted by the boundaries of more prescriptive models such as the medical or social model.^48^

### Poetry as an expression of psychosis

Whilst the importance of narratives has become increasingly ubiquitous in contemporary mental health discourse, observable in the emergence of specialities such as health humanities^14^ and narrative therapy^3^, the notion of narrative within the human experience is historically omnipresent. In essence, all poems are small stories ^49^, and Niles^50^ emphasizes that humans are natural storytellers, suggesting the term ‘homo narrans’ to emphasize the role that narratives play within society. In fact, it is perhaps the ability of humans to engage so enthusiastically with shared narratives as a way of defining lived time that is one of the defining attributes of human society.^51^

It is the primacy of narratives, and their potential to oppress and dominate lives, which is at the forefront of theory associated with narrative therapy.^3^ Whilst it could be argued that all therapy is a linguistic undertaking^34^, a narrative therapeutic philosophy hinges on the concept that narratives incorporate the process of ‘life making’, in which not only do we tell our stories, but we can become our stories.^51^ It is the construction and becoming of these narratives that remains paramount in the recovery process of individuals who have experienced mental illness.^52, 53^ However, it is also a breakdown in these stories, in the way in which narratives orientate us to the world, where the genesis of psychotic disorders might be found, arising from a time when the incongruence between our established stories and our objective reality becomes overwhelmingly distressing.^54^ It is in these psychotic states when the sense of self, which Parnas and Handest^55^ propose is inexorably intertwined with lived experience, is described as becoming diminished, no longer stable or undefined in some way.^55^

In reconstructing identity and narrative, those individuals who are living with psychosis may face great difficulties. Firstly in breaking free of the social poetics and stigma associated with a diagnosis,^56^ and secondly in identifying and utilising linguistic resources capable of conveying such complex subjective experiences.^57^ Therefore, a medium such as poetry which has the potential to resonate deeply with personal experience,^58^ whilst simultaneously offering individuals a wider range of linguistic tools,^59^ may be therapeutically beneficial. Moreover, the creation of narrative is not simply about the reconstruction of identity, but also represents a process of bearing witness to traumatic events, offering the potential for reintegration of the trauma within a sense of self.^60^ The use of poetry in such as way is encapsulated by the work of Hoeweller,^[61]^ who within his online blog, describes the use poetry has a tool to convey and understand the terror and complexity of his psychotic experience.

It is possible that the allure of poetry as a medium to convey such complex experiences arises from its potential to exist as what Bakhtin^62^ terms carnivalesque linguistics. Similar to the way in which those experiencing psychosis may create neologisms or play with words,^63^ carnivalesque language can be considered that which exists outside of established truth and order, and in some ways can be seen as a rebellion against the traditional confines of language.^64^ Furthering this notion of carnivalesque language, Bar-Am^[65]^ argues the importance of the notion of magical realism within the therapeutic sphere. This Literary genre, containing magical and supernatural events interwoven with objective reality, into personal narratives, has the potential to embrace elements of both poetry and psychotic experiences. Bar-Am^[65]^ suggests that this approach to understanding the elements of narrative cannot be understood within the confines of socially normalized structures, emphasizing that by positioning oneself within a post-modernist understanding of narratives one can engage with the experienced narrative as objective truth regardless of wider cultural norms. It is this full expression of a personal narrative which is key to the therapeutic process.^66^

However, one must consider if a personal narrative is ever entirely created or expressed by one author. As all utterances can be considered a response to previously encountered language,^62^ perhaps all narratives can be considered as originating from the dialogical and experiential rhizome. As we engaged in therapeutic meaning making dialogue, those involved may transition from being in the dialogue to being the dialogue, in which meaning is co-created.^68^ This notion or co-creation and co-narration is also commented on by Kristeva^[69]^ who suggests that upon starting narration, the subject of the narration becomes transformed into ‘neither nothingness nor anybody, but the possibilities of permutations’ (pp 74). In order for narration to commence, the individual must first experience emptiness, a blank space in which the subject is created in collaboration with the addressee. It is the individual, who through this process of narration, formulates themselves as both the subject within the narration and as the subject of the utterance. This process of self-destruction and self-creation, is one that is arguably omnipresent in mental health services as individuals seek not necessarily recovery, but rather discovery and post traumatic growth following mental illness.^70^

### Poetic exchange as therapeutic practice

The relationship between psychotherapy and poetry is one which had continues to evolve but appears inexorably linked.^71^ Freud is cited as saying that it is poets who discovered the unconscious and not psychoanalysts.^72^ This apparent relationship has led some to comment on the analogous qualities of both poetry and psychotherapy.

Amir^[73]^ suggests, using the terminology of music, that both poetry and psychotherapy work within the internal dialectic of tonal and atonal space within the psychic space of individuals. Wilkinson^[74]^ develops this notion further, suggesting that poetry and psychotherapy share three dimensions in which they overlap. Firstly, both are involved in experiences that are perhaps pre-communicable. Secondly, both have a relational dimension in which individuals reflect the way in which they relate with, and attach to others and their environment. Thirdly, both poetry and psychotherapy are rich in metaphor and symbolism. It is perhaps this last dimension, the one associated with meaning^74^, which is most relevant in consideration to the potential of poetry to support those who have experienced psychosis to understand their experiences. Moreover, it is this focus on meaning which emphasises the deeper exploration of language, beyond the immediately comprehendible.

Jacques Lacan was one of the first to operationalize semiotic theory in order to understand the metaphorical and symbolistic content within psychotherapy.^75^ The fundamental ontological position of semiotics is that language can and should be understood as a system of signs, expressed through the relationships between signifiers and signified.^76^ Signifiers are the words, sounds or images that generate the concept and the signified is the concept, which is generated. This semiotic process is occurring perpetually, in which all individuals are practising semioticians, reading signs in order to understand and interact with other people, culture and society.^76^ However, despite the structuralist origins of semiotic theory asserting that personal narratives are inescapably shaped by larger forces outside of the individual’s control. A distinction should be made between the system of signs which formulates language, some of which exist externally to the individual,^77,78^ and the process of meaning making which exists internally and is created within the mind of the individual.^79^

It is this meaning making process, and the semiotic content within the language utilized within the process, which has the potential to hold therapeutic value. For Lacan, it is the language which is primarily the resource for psychoanalysis, and so much attention must be devoted to the analysand’s use of metaphor and metonymy, the exchange of one word for another. This use of language, potentially driven by the Freudian mechanisms of condensation and displacement,^80^ represents more than simply a choice of words, but rather signifies how one sees the world.^81^ Furthermore, it is a breakdown in this semiotic process that results in the development of psychotic phenomena. Kristeva^[69]^ goes further and suggests that psychosis arises from ‘the borders where signification vanishes’. Herein, the authors suggest that if or when language fails to contain an experience or phenomena, individuals can be pulled into a psychic void symbolized as semiotic chaos.

This chaos may also be observed in the way, during psychosis, the use of metaphor may shift from figurative representation to more concrete experience as an individual struggles to distinguish between thoughts, feelings and perceptions^82^. This articulation of metaphors in literal terms may, in turn, result in the metaphor being heard literally by the addressee.^83^ However, in order to understand these experiences, one must build a bridge between the idiosyncratic meaning produced by the individual and the broader shared understanding of the words and metaphors within a broader culture or environment.^83,84^ This position of emphasizing the importance of looking beyond the initial experience of the language is also evident within poetry. Riffaterre^79^ suggests that when working with poetry one must first hurdle the obstacle of mimesis, rejecting the initial, obvious relationship of words to reality, in order to search for deeper, perhaps more esoteric meanings. This approach of searching for the more opaque meanings, may require an acceptance of different realities existing simultaneously as experienced by different individuals.^85^

Whilst this ontological underpinning has the potential to offer a more therapeutically responsive dialogue, as it has no agenda and embraces uncertainty, one must also consider the potential for the therapeutic space to become distorted due to a lack of boundaries. An engagement with poetry, just as in psychotherapy, is an enactment, an unconscious process undertaken by two individuals.^74^ Therefore, there is the potential for the words and experiences of the author and reader/ interlocutor to become intertwined^86^ or for the reader/ interlocutor to annotate the poem and the subsequent meanings as a result of their own experiences and.^87,88^ It is in these moments that both parties may succumb to the allure of the affective fallacy, whereby individuals may conflate the poem or poetry itself and their own emotive response to that piece of poetry.^89^

## Conclusions: Limitations and Implications

### Relationship to prior work

The conceptual review outlined in this paper, and its triadic domains propose a relationship between poetry and psychosis consisting of three domains. The framework consists of three domains: psychotic language as meaningful language, poetry as an expression of psychosis, and psychotherapeutic practice and poetry. This novel conceptualization represents a departure from traditional biomedical paradigms and a refocusing on the significance of individual narratives and poetics, proposing that not only can psychosis be understood in terms of poetics, but also that such poetic undertakings might hold significant therapeutic value.^1^ Lucas^[90]^ proposes that in order to fully understanding psychotic communication, one must become tuned into the psychotic wavelength. The domains identified within this paper perhaps develop this notion further, proposing a poetic wavelength. A wavelength upon which both narrator and interlocutor are able to utilize poetry to narrate, understand and make meaning of psychotic experiences.

Poets have long wrestled with their tormenting muses,^6,91^ and psychosis should not be venerated as a source of poetic inspiration. However, if psychosis, can be considered a form of poetry, in which the use of metaphor and metonymy are purposeful and not disordered,^81^ then perhaps the need for, and focus on, coherence is reduced. Moreover, this fresh assessment of psychotic speech enables the narrator and interlocutor to engage in potential semiotic, psychological and linguistic processes in a more inquisitive manner, accepting the uncertainty and seeking dialogue.

There is an acceptance that the restoration of personal identity is crucial in the recovery process following psychosis.^92^ It is often through the construction of narratives that individuals can arrive at such an understanding of their experiences and identity.^51^ However, the limitations of formal language may restrict the ability of an individual to adequately express complex experiences such as psychosis.^57^ However, poetry with its metaphor and semiotic laden stanzas may offer a medium through which an individual can not only craft their narrative but also shift the discourse into a form which holds the most meaning for them.^93^

### Strengths and limitations

The initial searches undertaken in order to identify literature were not undertaken in an attempt to identify all literature associated with the topic, as would be the case in a systematic review. Rather, the searches aimed to review was to synthesise literature from a range of topics across a range of disciplines. However, it could be argued that the search terms used were rather broad and specific seminal pieces of literature might have been missed. Furthermore, although the research involved all three members of the team, the initial searches and primary synthesis was undertaken by one member of the team, which again, might have increased the potential for literature to be missed during the search process.

The search for grey literature also aimed to capture some of the voices of those with lived experience of psychosis. However, there is a limitation to how generalizable the experience of people is who document their experiences online, especially considering the idiosyncratic and complex phenomenon of psychosis. Furthermore, there was no representative service user or carer collaboration in the development of the conceptual framework which might have further increased validity.^94^

### Implications for practice

The majority of contemporary mental healthcare services, especially within western Europe and North America, despite the identified shortfalls in this model,^95^ continue to rely on the biomedical paradigm when working with those who are described as experiencing psychosis.^96^ However, for many individuals, this biomedical understanding of their experiences lacks meaning and may be further stigmatizing.^13, 97^ Therefore, this conceptual framework seeks to advance the discourse in relation to psychosis, considering novel ways in which this phenomenon can be understood. It is the purpose of conceptual reviews to not only systematically identify existing phenomena but also to guide further enquiry within the empirical world.^98^ Therefore, the conceptual framework presented in this paper might serve to act as a foundation for further research, providing a conceptual orientation to guide further enquiry into the therapeutic potential of poetry for those who have experienced psychosis. Moreover, psychosis remains a complex phenomenon, especially in relation to how mental health services can effectively support those who have experienced psychosis in their recovery. For those working therapeutically with individuals who have or are experiencing psychosis this conceptual framework might provide the basis for further poetic enquiry within their work, enabling a more polyphonic approach to understanding psychosis, which see past the immediate meaning of language, hurdle mimesis and consider the poetics of psychosis. Whilst the conceptual review does not prescribe specific activities to be undertaken by practitioners, it does provide an initial orientation to the way in which language and poetry could be utilised therapeutically within mental health services and therapy more broadly.

## Figures and Tables

**Table 1:** Conceptual review domains

Conceptual review domains	Sub themes within domain
**Psychotic Language as meaningful poetics**	Meaning within psychotic experiences
	Psychosis as a medium of expression
	Psychosis as an exercise in linguistic meaning making
	Coherence within poetics
**Poetry as an expression of psychosis**	Narratives as central to identity and understanding experiences
	Restricted narrative expression for those who have experienced psychosis
	Poetry as carnivalesque language
	The potential for co-narration of self with others
**Poetic Exchange as therapeutic practice**	Analogous relationships between poetry and psychotherapy
	Language as key to understanding
	Psychosis as a semiotic process
	Metaphors as a bridge between external and internal realities
